# Foveal Curvature and Its Associations in UK Biobank Participants

**DOI:** 10.1167/iovs.63.8.26

**Published:** 2022-07-28

**Authors:** Abraham Olvera-Barrios, Yuka Kihara, Yue Wu, Alasdair N. Warwick, Philipp L. Müller, Katie M. Williams, Alicja R. Rudnicka, Christopher G. Owen, Aaron Y. Lee, Catherine Egan, Adnan Tufail, on behalf of the UK Biobank Eyes and Vision Consortium

**Affiliations:** 1 Medical retina, Moorfields Eye Hospital NHS Foundation Trust, London, United Kingdom; 2 Institute of Ophthalmology, University College London, London, United Kingdom; 3 Roger and Angie Karalis Johnson Retina Center, University of Washington, Seattle, WA, United States; 4 Department of Ophthalmology, School of Medicine, University of Washington, Seattle, WA, United States; 5 Institute of Cardiovascular Science, University College London, London, United Kingdom; 6 Macula Center, Südblick Eye Centers, Augsburg, Germany; 7 Department of Ophthalmology, University of Bonn, Bonn, Germany; 8 Section of Ophthalmology, School of Life Course Sciences, FoLSM, King's College London, United Kingdom; 9 Population Health Research Institute, St. Georges, University of London, London, United Kingdom

**Keywords:** retina, fovea, optical coherence tomography, OCT, development, UK Biobank

## Abstract

**Purpose:**

To examine whether sociodemographic, and ocular factors relate to optical coherence tomography (OCT)–derived foveal curvature (FC) in healthy individuals.

**Methods:**

We developed a deep learning model to quantify OCT-derived FC from 63,939 participants (age range, 39–70 years). Associations of FC with sociodemographic, and ocular factors were obtained using multilevel regression analysis (to allow for right and left eyes) adjusting for age, sex, ethnicity, height (model 1), visual acuity, spherical equivalent, corneal astigmatism, center point retinal thickness (CPRT), intraocular pressure (model 2), deprivation (Townsend index), higher education, annual income, and birth order (model 3). Fovea curvature was modeled as a z-score.

**Results:**

Males had on average steeper FC (0.077; 95% confidence interval [CI] 0.077–0.078) than females (0.068; 95% CI 0.068–0.069). Compared with whites, non-white individuals showed flatter FC, particularly those of black ethnicity. In black males, −0.80 standard deviation (SD) change when compared with whites (95% CI −0.89, −0.71; *P* 5.2e10^−68^). In black females, −0.70 SD change when compared with whites (95% CI −0.77, −0.63; *p* 2.3e10^−93^). Ocular factors (visual acuity, refractive status, and CPRT) showed a graded inverse association with FC that persisted after adjustment. Macular curvature showed a positive association with FC. Income showed a linear trend increase in males (*P* for linear trend = 0.005).

**Conclusions:**

We demonstrate marked differences in FC with ethnicity on the largest cohort studied for this purpose to date. Ocular factors showed a graded association with FC. Implementation of FC quantification in research and on the clinical setting can enhance the understanding of clinical macular phenotypes in health and disease.

The fovea is a highly specialized retinal region at the center of the macula responsible for driving high visual acuity and color vision.^1^ Despite occupying ∼2.69 mm^2^/1100 mm^2^ of the retinal area, the fovea maps to half of the primary visual cortex.^2^^,^^3^

Absent or poorly formed foveal depressions with presence of inner retinal layers have been associated with poor vision in cases with well characterized diseases (i.e., retinopathy of prematurity, aniridia, ocular albinism, absent or poorly formed foveal depressions with presence of inner retinal layers optic nerve decussation defects and anterior segment dysgenesis syndrome, Stickler syndrome, Alport syndrome, familial exudative vitreoretinopathy, incontinentia pigmenti, nanophthalmos, posterior microphthalmos, and achromatopsia).^4^^–^^10^ Nevertheless, absent foveas or foveas with presence of inner retinal layers have also been described in healthy individuals with good vision.^11^ In this context, the detailed noninvasive cross-sectional imaging of the retina with micrometer resolution obtained with optical coherence tomography (OCT) has significantly contributed to the detailed quantitative description of foveal morphology in healthy and diseased individuals and provided insight into postnatal retina development.^12^^–^^14^ Our understanding of mechanisms and functional implications of cytoarchitectural and morphological foveal alterations are driven by studies in patients with absent or poorly formed foveal depressions with presence of inner retinal layers in selective settings.^15^ What is less understood is the interindividual variation of foveal curvature (FC) in the general population, and what factors may be associated with these differences. Studies analyzing the OCT-derived foveal slope have been limited to using small to moderate sample sizes (typically with less than 400 subjects).^6^^,^^16^^–^^19^

With more than half a million recruited participants and with a subset of about 85,000 patients with enhanced ophthalmological examination, the UK Biobank is one of the world's largest single resources for comprehensive study of health and disease.^20^ By using comprehensive structured population data and machine learning (ML), we aim to address a gap in our knowledge by exploring the associations of sociodemographic, ocular, and early life factors with OCT-derived FC of healthy individuals.

## Methods

### Study Population

The UK Biobank is a national research resource with the aim of improving the prevention, diagnosis, and treatment of a wide range of diseases.^9^ The study recruited more than 500,000 people aged 40 to 69 between 2006 to 2010 from across the United Kingdom. Ethical approval was obtained from the Northwest Region National Health Service research ethics committee (REC reference number 06/MRE08/65), and all participants provided written informed consent.

### Ophthalmic Examination Protocol

The design and methods in the UK Biobank Eye and Vision Consortium have been published elsewhere.^21^ More than 133,000 participants underwent an enhanced ophthalmic assessment. A subset of these (87,624 participants) had undilated macular spectral-domain OCT (SD-OCT, Topcon 3D OCT-1000; Topcon Optical Company, Tokyo, Japan) imaging.

#### Spectral-Domain Optical Coherence Tomography Imaging Protocol

Undilated SD-OCT imaging was carried out with the Topcon 3D OCT-1000 Mark II (Topcon Optical Company) using the three-dimensional 6 × 6 mm^2^ volume scan mode (128 B-scans with 512 A-scans per eye). Imaging was performed after visual acuity, noncycloplegic autorefraction, and intraocular pressure (IOP) measurement. The right eye was scanned first.

### Automated Foveal Parameter Analysis

#### RPE-ILM Boundary Segmentation

**Figure 1.**
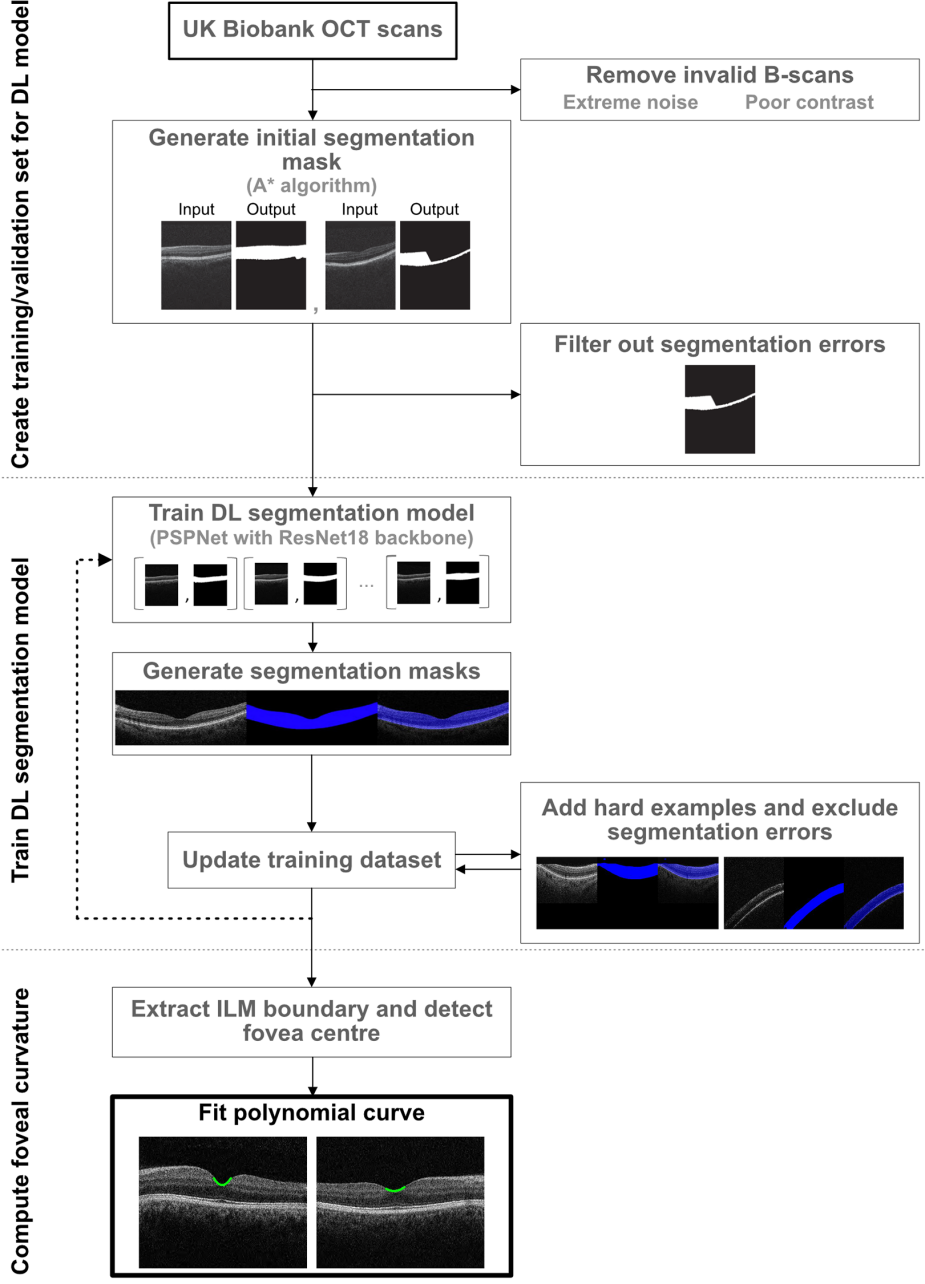
Diagram of machine learning methodology. DL, deep learning.

Figure 1 shows the methodology implemented to generate our automated OCT-derived FC quantification (Supplementary Material). We developed a ML model to extract the area between the internal limiting membrane (ILM) and retinal pigment epithelium (RPE) from OCT B-scans and detect ILM boundaries without need for human annotations. We used the A star (A*) algorithm^22^ to obtain initial layer segmentation masks to be used as training targets. A collected sample of 6409 input-output pairs was split into training (80%) and validation sets (20%) containing mutually exclusive groups of subjects. We used the Pyramid Parsing Network with a ResNet-18 backbone as our segmentation architecture.^23^^,^^24^ The model achieved a mean intersection over union score = 0.97 on the validation set. Last, ILM boundaries were extracted by tracking the top boundary for each segmentation mask.

#### Center Point Retinal Thickness Analysis

Center point retinal thickness (CPRT) was determined to be the center of the area with the thinnest retina for each OCT volume scan and its location given as a tuple of slice number and B-scan x-coordinate (With higher coefficients meaning steeper FC, Supplementary Fig. S1). The retinal thickness in pixels was computed as the difference between the ILM and RPE y-coordinates (obtained from segmentation masks) at the fovea center point.

#### Foveal Curvature Fitting

The curve fitting was executed on the pixel coordinates. Given the center point for each OCT volume scan, a two-dimensional polynomial curve was fitted on the extracted ILM boundary with a range of 12 pixels left and right from the center point. The degree of the polynomial used was two dimensional, and the coefficient of highest degree (leading term) was used to describe the curvature (Fig. 1).

#### Macular Curvature Fitting

We used 32 central B-scans for macular curvature fitting. For each B-scan, we fitted a quadratic function to the extracted RPE boundary, then took the coefficient of the leading term as curvature value. After collecting the 32 curvature values, we took median as the final macular curvature of the volume. Again, the curve fitting was executed on the pixel coordinates (Supplementary Fig. S2).

### Validation of Automated Foveal Curvature Analysis

Two retina specialists with wide experience in OCT grading (A.T., A.O-B.) were asked to classify 10 different image sets, composed of 3 B-scans from each FC tertile (see Supplementary Material and Supplementary Fig. S3), from flattest to steepest scan in each set. The reference standard was a tertile classification based on ML-derived FC quantification. Human graders correctly classified each FC tertile in all 10 image sets.

### Other Covariates

Data obtained from the touchscreen questionnaire including information about sociodemographic factors (age, sex, self-reported ethnicity, educational or professional qualification, income), early life factors (birth order, birth weight, breastfed as a baby), and family history (mother's age at birth, number of siblings, maternal smoking around birth) were collected. Ethnicity was coded as white, black or black British, Asian or Asian British, mixed, Chinese, other ethnic group, do not know, and prefer not to answer.^25^ Height was obtained from the physical measures category (Data-field 50, https://biobank.ctsu.ox.ac.uk/crystal/field.cgi?id=50). Best-corrected visual acuity (VA), in logarithm of minimum angle of resolution, autorefractor-derived diopters (D) of spherical equivalent (SE) and corneal astigmatism (in D, calculated as steepest curvature K_max_ − flattest curvature K_min_), as well as IOP (mm Hg measured by Goldman applanation tonometry) measurements from the ocular examination were included in the analysis. Axial length (AL) measurement was not part of the UK biobank examination protocol and thus was not available for analysis. The fluid intelligence score was additionally included as a cognitive indicator. Deprivation was expressed as Townsend deprivation index at neighborhood postcode level.

### Inclusion and Exclusion Criteria

**Figure 2.**
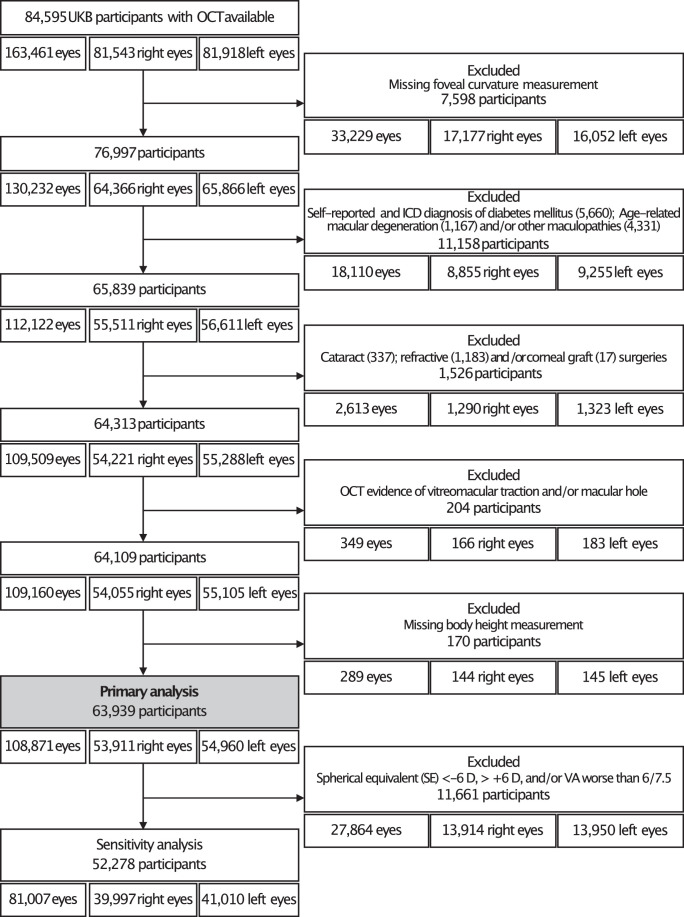
Participants included in the study. UKB; UK biobank, OCT; Optical coherence tomography, ICD; International classification of diseases, D; Diopter, VA; Visual acuity.

A flowchart of participants by exclusions is shown in Fig. 2. Eyes of participants with self-reported age-related macular degeneration, diabetic retinopathy, macular disease, and eyes with cataract, refractive and corneal graft surgeries were excluded from the analysis.

### Statistical Analysis

R version 4.0.2 was used to analyze the data.^26^ The “lme4” (version 1.1-28) package was used for linear multilevel regression models fitted by restricted maximum likelihood.^27^ *P* values were calculated via Satterthwaite's degrees of freedom method with the “lmerTest” (version 3.1-3) package.^28^ In view of systematic differences in FC between men and women, and associated covariates related to growth, all analyses were stratified by sex. Multilevel linear regression models adjusting for age, ethnicity, height, and UK Biobank assessment center as fixed effects, with a random effect for person to allow for the right- and left-eye data from the same participant to contribute to the analysis (Model 1), were used to examine associations with FC. Model 2 extended model 1 with further adjustment for VA, SE, corneal astigmatism, IOP, MC, and CPRT. Model 3 extended model 2 allowing for deprivation, higher education, fluid intelligence score, annual income, and birth order. Fovea curvature measures were modeled as z scores. Coefficients represent the standard deviation (SD) change in FC per specified increase in covariates or the standardized difference between groups (Supplementary Tables S1 and S2, show estimates for raw FC × 100 as dependent variable). Data missing on categorical variables were included as an additional category for each variable to minimize data loss. In sensitivity analyses model 3 was extended by allowing for birth weight, maternal age at birth, maternal smoking around birth, and breastfeeding status as a baby to examine FC associations with early life factors. Additionally, multilevel models were fitted again after exclusion of individuals with SE <−6 D and >6 D and vision <80 Early Treatment Diabetic Retinopathy Study letters (worse than 6/7.5 Snellen, or worse than 0.1 logMAR equivalent).

## Results

**Figure 3.**
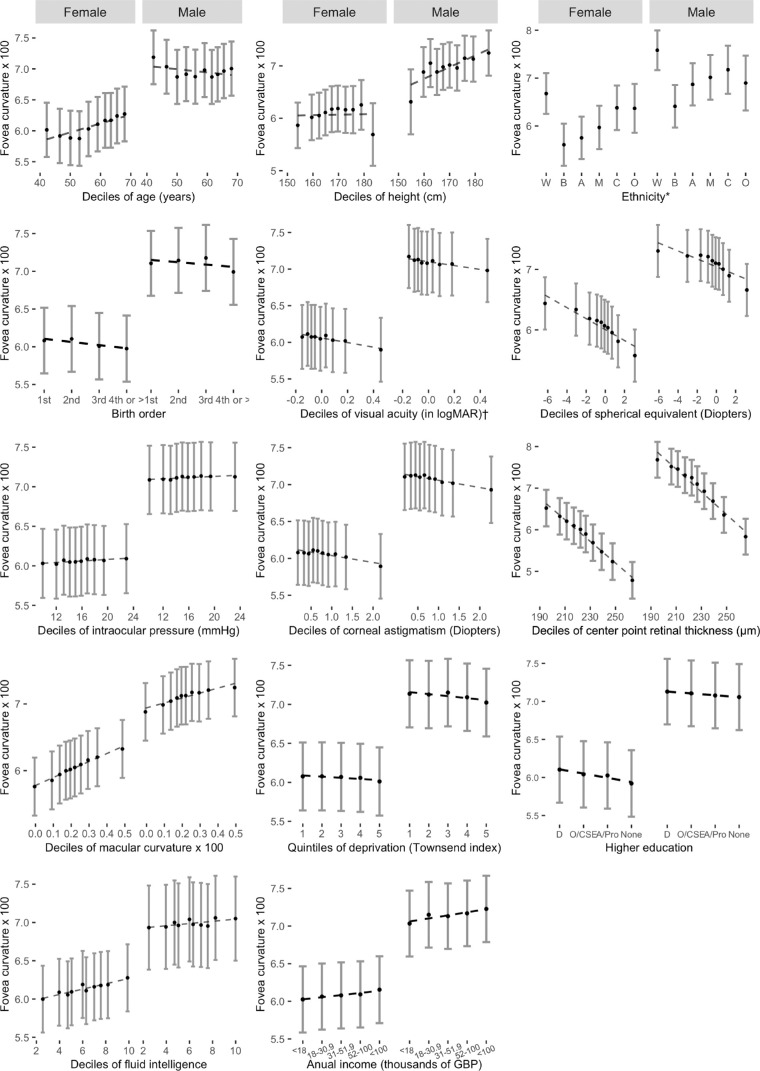
Adjusted mean foveal curvature by deciles of covariates stratified by sex (annual income and Townsend index of deprivation shown in quintiles). Adjusted means (*solid* *black dots*), 95% confidence intervals (*vertical* *solid lines*), and regression line (*dotted* *line*) are from a multilevel model allowing for age, height, ethnicity, and UK Biobank center as fixed effects, and repeated foveal curvature measurement for each person. *Ethnicity codes: W, white; B, black; A, Asian; M, mixed; C, Chinese; O, other. † Visual acuity shown in logMAR for visualization purposes. GBP, pound sterling; O/CSE, O levels, certificate of secondary education or equivalent.

Table 1 shows the overall patient characteristics of our study cohort. A summary of eye-level characteristics is found in Table 2. A total of 109,160 eyes (54,055 right eyes, and 55,105 left eyes) of 63,939 participants (45.1% male) were included in the analysis. Mean age (SD) was 56 years (±8.0), and 92% of the participants were White. The FC followed a normal distribution (Supplementary Fig. S3) and had a mean of 0.072 (±0.02). Figure 3 shows the association of FC with each covariate (deciles of continuous variables), adjusted for age, height, and UK Biobank assessment center. Foveal curvature showed an inverse association with each decile increase in SE, CPRT, VA, and corneal astigmatism. A positive linear association of FC was found for each decile increase in MC. Associations in different directions between males and females were observed with age. Associations were less clear for height, IOP, higher education, fluid intelligence, annual income, and deprivation.

**Table 1.** Patient Level Characteristics Stratified by Sex

Characteristic	Overall (N = 63,939^*^)	Female (N = 35,097^*^)	Male (N = 28,842^*^)
Age	56 (8.0)	56 (7.9)	56 (8.2)
Ethnicity			
White	58,915 (92.1%)	32,204 (91.8%)	26,711 (92.6%)
Black	1,614 (2.5%)	979 (2.8%)	635 (2.2%)
Asian	1,477 (2.3%)	767 (2.2%)	710 (2.5%)
Other	822 (1.3%)	486 (1.4%)	336 (1.2%)
Mixed	521 (0.8%)	339 (1.0%)	182 (0.6%)
Chinese	248 (0.4%)	153 (0.4%)	95 (0.3%)
Prefer not to say	226 (0.4%)	109 (0.3%)	117 (0.4%)
Missing	116 (0.2%)	60 (0.2%)	56 (0.2%)
Height (cm)	169 (9.2)	163 (6.3)	176 (6.7)
Townsend deprivation quintiles			
1	20,388 (31.9%)	10,995 (31.3%)	9,393 (32.6%)
2	13,277 (20.8%)	7,266 (20.7%)	6,011 (20.8%)
3	11,828 (18.5%)	6,618 (18.9%)	5,210 (18.1%)
4	11,170 (17.5%)	6,304 (18.0%)	4,866 (16.9%)
5	7,204 (11.3%)	3,877 (11.0%)	3,327 (11.5%)
Missing	72 (0.1%)	37 (0.1%)	35 (0.1%)
Income (in GBP)			
Less than 18,000	9,802 (15.3%)	5,696 (16.2%)	4,106 (14.2%)
18,000 to 30,999	13,569 (21.2%)	7,584 (21.6%)	5,985 (20.8%)
31,000 to 51,999	15,261 (23.9%)	8,061 (23.0%)	7,200 (25.0%)
52,000 to 100,000	13,530 (21.2%)	6,745 (19.2%)	6,785 (23.5%)
Greater than 100,000	4,431 (6.9%)	2,138 (6.1%)	2,293 (8.0%)
Prefer not to say	7,104 (11.1%)	4,741 (13.5%)	2,363 (8.2%)
Missing	242 (0.4%)	132 (0.4%)	110 (0.4%)
Education			
Degree	23,875 (37.3%)	12,729 (36.3%)	11,146 (38.6%)
O levels, CSEs, or equivalent	15,840 (24.8%)	9,227 (26.3%)	6,613 (22.9%)
A levels, professional, or equivalent	13,730 (21.5%)	7,344 (20.9%)	6,386 (22.1%)
None	8,256 (12.9%)	4,350 (12.4%)	3,906 (13.5%)
Missing	2,238 (3.5%)	1,447 (4.1%)	791 (2.7%)
Fluid intelligence^†^	6 (2.1)	6 (2.1)	6 (2.2)
Birth order			
1	35,661 (55.8%)	19,803 (56.4%)	15,858 (55.0%)
2	20,216 (31.6%)	10,855 (30.9%)	9,361 (32.5%)
3	4,034 (6.3%)	2,237 (6.4%)	1,797 (6.2%)
4	3,989 (6.2%)	2,182 (6.2%)	1,807 (6.3%)
Missing	39 (0.1%)	20 (0.1%)	19 (0.1%)

GBP, pound sterling; CSE, certificate of secondary education.

* Mean (SD) for continuous variables; n (%) for categorical variables.

† Continuous variable with missing data (2.7%).

**Table 2.** Eye Level Characteristics Stratified by Sex

		Sex^*^	
Characteristic	Overall^*^N = 108,871^†^	Female, N = 59,642^†^	Male, N = 49,229^†^
Fovea curvature × 100	7.25 (7.24, 7.27)	6.83 (6.82, 6.85)	7.77 (7.75, 7.78)
Visual acuity (in ETDRS letters)	85.09 (85.03, 85.14)	84.78 (84.70, 84.85)	85.46 (85.37, 85.55)
Spherical equivalent (Diopter)	−0.69 (−0.70, −0.67)	−0.71 (−0.73, −0.69)	−0.66 (−0.69, −0.64)
Corneal astigmatism (Diopter)	0.85 (0.85, 0.85)	0.88 (0.88, 0.89)	0.81 (0.80, 0.81)
Macula curvature x 100	0.21 (0.21, 0.22)	0.21 (0.21, 0.22)	0.22 (0.21, 0.22)
Center point retinal thickness (in µm)	226.18 (226.06, 226.30)	223.11 (222.96, 223.26)	229.90 (229.72, 230.08)

ETDRS, Early Treatment Diabetic Retinopathy Study.

* N eyes of 63,939 participants.

† Mean (95% CI).

### Sex Differences

Males had on average steeper FC and greater CPRT. Crude difference in FC between men and women was 0.47 SDs, and after adjustment for age, ethnicity, height, and UK biobank assessment center (i.e., as in model 1), this difference was 0.36 SDs (95% CI 0.34–0.38; *p* 4.8 × 10^−247^). After additional adjustment for covariates included in model 3 (SE, VA, IOP, MC, CPRT, corneal astigmatism, higher education, annual income, fluid intelligence, deprivation, and birth order) the sex-difference was 0.46SD (95% CI 0.44–0.48; *p* 4.3 × 10^−360^). Formal tests for interaction with sex (females vs. males from multilevel models adjusting for age, sex, height, and UK biobank center as fixed effects, and a random effect per person) were significant for age, MC and CPRT only (all instances *P* < 0.0001). As a result of the observed systematic sex differences, associations are presented for females (Table 3) and males (Table 4) separately.

**Table 3.** Standardized Difference in Fovea Curvature Per Specified Differences in Covariates ([95% CI]; p-Value) for Females.

Characteristic	Females Model 1*	Females Model 2^†^	Females Model 3^‡^
Age (Per Decade)	0.07 (0.06, 0.08); **6.8e-28**	0.08 (0.07, 0.09); **1.8e-34**	0.08 (0.07, 0.10); **9.4e-30**
Ethnicity			
White	1.00	1.00	1.00
Black	−0.54 (−0.59, −0.48); **5.1e-75**	−0.73 (−0.79, −0.67); **1.8e-120**	−0.70 (−0.77, −0.63); **2.3e-93**
Asian	−0.46 (−0.53, −0.40); **2.2e-44**	−0.59 (−0.66, −0.52); **1.4e-63**	−0.56 (−0.63, −0.48); **9.9e-48**
Other	−0.35 (−0.43, −0.27); **3.8e-18**	−0.45 (−0.53, −0.36); **4.4e-25**	−0.40 (−0.49, −0.31); **8.2e-18**
Mixed	−0.15 (−0.24, −0.05); **0.002**	−0.26 (−0.35, −0.16); **3.7e-07**	−0.24 (−0.34, −0.13); 7.5e-06
Chinese	−0.15 (−0.30, −0.01); **0.032**	−0.32 (−0.46, −0.17); **2.5e-05**	−0.30 (−0.45, −0.14); **1.7e-04**
Prefer not to say	−0.42 (−0.59, −0.26); **6.9e-07**	−0.51 (−0.69, −0.33); **1.7e-08**	−0.52 (−0.72, −0.33); **9.5e-08**
Missing	−0.41 (−0.63, −0.18); **3.5e-04**	−0.44 (−0.68, −0.20); **3.1e-04**	^§^
Height (per 5 cm)	0.04 (0.03, 0.05); **5.5e-24**	0.04 (0.03, 0.04); **2.4e-19**	0.03 (0.03, 0.04); **6.9e-16**
Visual acuity (per 5 letters)		0.01 (0.01, 0.02); **1.6e-16**	0.01 (0.01, 0.02); **1.4e-15**
Spherical equivalent (per diopter)		−0.04 (−0.05, −0.04); **6.4e-105**	−0.04 (−0.04, −0.04); **1.3e-96**
Corneal astigmatism (per diopter)		−0.07 (−0.08, −0.06); **3.2e-33**	−0.07 (−0.08, −0.06); **4.0e-32**
Macula curvature (per 0.01)		0.29 (0.23, 0.35); **1.4e-21**	0.28 (0.22, 0.34); 1.7e-19
Center point retinal thickness (per 10 µm)		−0.13 (−0.13, −0.13); **0.0e+00**	−0.13 (−0.14, −0.13); **0.0e+00**
Fluid intelligence			0.01 (0.00, 0.01); **9.8e-04**
Annual income (Great British Pound)			
Less than 18,000			1.00
18,000 to 30,999			0.01 (−0.03, 0.04); 0.701
31,000 to 51,999			0.01 (−0.02, 0.04); 0.589
52,000 to 100,000			0.01 (−0.03, 0.05); 0.605
Greater than 100,000			0.03 (−0.02, 0.08); 0.181
Prefer not to say			0.04 (0.00, 0.07); 0.055
Missing			−0.02 (−0.27, 0.23); 0.872
Per increase in income category			0.02 (−0.01, 0.06); 0.230
Birth order			
1			1.00
2			0.01 (−0.01, 0.03); 0.311
3			−0.02 (−0.06, 0.02); 0.259
4			−0.01 (−0.06, 0.03); 0.569
Missing			0.19 (−0.23, 0.60); 0.371

Bold *P* values represent statistically significant results.

* Model 1: multilevel model adjusts for age, ethnicity, and height as fixed effects, and a random effect for person to allow for within person eye measurements (59,642 eyes of 35,097 patients).

† Model 2 adjusts as model 1 plus visual acuity, spherical equivalent, corneal astigmatism, macular curvature, and center point foveal thickness as fixed effects (54,489 eyes of 32,564 patients).

‡ Model 3 adjusts as model 2 plus deprivation, higher education, fluid intelligence score, annual income, and birth order as fixed effects (53,135 eyes of 31,727 patients).

§ No missing data on ethnicity on this model.

**Table 4.** Standardized Difference in Fovea Curvature Per Specified Differences in Covariates ([95% CI]; p-Value) for Males.

Characteristic	Males Model 1^*^	Males Model 2^†^	Males Model 3^‡^
Age (per decade)	−0.03 (−0.04, −0.01); **8.4e-05**	−0.01 (−0.03, 0.00); 0.168	−0.01 (−0.02, 0.01); 0.466
Ethnicity			
White	1.00	1.00	1.00
Black	−0.59 (−0.67, −0.51); **6.0e-49**	−0.84 (−0.92, −0.76); **2.1e-88**	−0.80 (−0.89, −0.71); **5.2e-68**
Asian	−0.36 (−0.43, −0.28); **7.4e-21**	−0.54 (−0.62, −0.46); **1.7e-41**	−0.54 (−0.62, −0.45); **2.3e-34**
Other	−0.28 (−0.39, −0.18); **1.5e-07**	−0.48 (−0.60, −0.37); **1.5e-17**	−0.47 (−0.59, −0.35); **7.2e-15**
Mixed	−0.20 (−0.35, −0.06); **0.005**	−0.38 (−0.53, −0.24); **3.8e-07**	−0.37 (−0.52, −0.21); **3.0e-06**
Chinese	−0.34 (−0.54, −0.15); **6.5e-04**	−0.53 (−0.73, −0.32); **4.5e-07**	−0.54 (−0.76, −0.33); **8.9e-07**
Prefer not to say	−0.13 (−0.31, 0.05); 0.150	−0.13 (−0.31, 0.05); 0.168	−0.09 (−0.29, 0.11); 0.372
Missing	0.00 (−0.25, 0.26); 0.986	−0.04 (−0.31, 0.22); 0.749	−0.54 (−1.9, 0.82); 0.437
Height (per 5 cm)	0.04 (0.03, 0.05); **4.3e-19**	0.04 (0.03, 0.04); **3.4e-15**	0.03 (0.02, 0.04); **4.2e-12**
Visual acuity (per 5 letters)		0.01 (0.01, 0.01); **3.5e-07**	0.01 (0.01, 0.01); **8.4e-07**
Spherical equivalent (per diopter)		−0.04 (−0.04, −0.03); **5.3e-53**	−0.04 (−0.04, −0.03); **5.6e-50**
Corneal astigmatism (per diopter)		−0.06 (−0.08, −0.05); **3.6e-20**	−0.06 (−0.08, −0.05); **5.3e-20**
Macula curvature (per 0.01)		0.14 (0.07, 0.21); **1.0e-04**	0.14 (0.07, 0.21); **1.2e-04**
Center point retinal thickness (per 10 µm)		−0.14 (−0.15, −0.14); **0.0e+00**	−0.15 (−0.15, −0.14); **0.0e+00**
Fluid intelligence			0.00 (−0.01, 0.00); 0.559
Annual income (Great British Pound)			
Less than 18,000			1.00
18,000 to 30,999			0.05 (0.01, 0.10); **0.010**
31,000 to 51,999			0.05 (0.01, 0.09); **0.021**
52,000 to 100,000			0.07 (0.02, 0.11); **0.004**
Greater than 100,000			0.08 (0.02, 0.14); **0.005**
Prefer not to say			0.00 (−0.05, 0.06); 0.857
Missing			0.11 (−0.18, 0.41); 0.459
Per increase in income category			0.06 (0.02, 0.10); **0.005**
Birth order			
1			1.00
2			0.00 (−0.02, 0.03); 0.728
3			0.03 (−0.02, 0.08); 0.216
4			−0.02 (−0.08, 0.03); 0.375
Missing			−0.20 (−0.65, 0.25); 0.389

Bold *P* values represent statistically significant results.

* Model 1: multilevel model adjusts for age, ethnicity, and height as fixed effects, and a random effect for person to allow for within person eye measurements (49,229 eyes of 28,842 patients).

† Model 2 adjusts as model 1 plus visual acuity, spherical equivalent, corneal astigmatism, macular curvature, and center point foveal thickness as fixed effects (45,296 eyes of 26,982 patients).

‡ Model 3 adjusts as model 2 plus deprivation, higher education, fluid intelligence score, income, and birth order as fixed effects (44,118 eyes of 26,252 patients).

### Age, Ethnicity, and Height (Models 1 to 3)

Differences in FC by ethnicity showed the greatest effect overall, and these held after adjustment, and after exclusion of extreme refractive status and low VA. Black, Asian, mixed, Chinese, and other ethnic groups showed flatter FC when compared with whites. Black participants showed the biggest difference (In models 3, males 0.80SD decrease [*p* 2.1 × 10^−88^]; females 0.70 SD decrease when compared to whites [*p* 1.8 × 10^−120^]).

In females, every decade increase in age was associated with a 0.07 SD rise in FC (Model 1; *p* 6.8 × 10^−28^), and additional adjustments, or exclusion of those on basis of high SE, did not materially alter the strength of this association (Supplementary Table S3). In males, a 0.03 SD decline in FC per decade rise in age was observed in Model 1, but this was attenuated to the null with further adjustment (Models 2 and 3, Table 4).

Every 5 cm increase in height was associated with steeper foveas in males and females, with equal effect sizes across all models in both sexes. Formal test for interaction between ethnicity with age, height, and SE showed that patterns were consistent across ethnicity (data available on request) except for the age in females. Analyses showed that for female non-white ethnicities there was no association with age but the rise in FC with age was present in white females only (0.10 SD increase in FC per decade rise in age; 95% CI 0.09–0.11)

### Functional and Ocular Factors (Models 2 and 3)

Every five Early Treatment Diabetic Retinopathy Study letter increase in VA (better vision) was associated with a 0.01 SD rise in FC in both males and females in all models (*p* in all instances ≤3.5 × 10^−7^). Every D increase in corneal astigmatism and in SE was associated with a flatter FC in both males and females. An inverse association was observed with foveal thickness in both sexes, per 10µm increase in foveal thickness FC decreased by approximately 0.1 SD. Steeper MC were found to be associated with steeper FC measurements and the effect sizes were double in females when compared with males in all models. After exclusion of extreme refractive status and VA worse than 6/7.5, the association with MC was attenuated to the null in males (Supplementary Table S4) but did not materially change in females. IOP did not show an association with FC.

### Additional Sociodemographic Factors (Model 3)

Fovea curvature showed an increasing trend in curvature steepness with increasing annual income in males (*p* for linear trend 0.005). When compared to annual income <18,000 GBP, earning >100,000 GBP per year was associated with a 0.08 SD rise in FC in males (Model 3, *p* for linear trend 0.005). The FC associations with income were not observed in females.

Fluid intelligence showed a significant 0.01 SD rise in FC per score unit increase (95% CI 0.002–0.099; *p* 9.8 × 10^−4^) in females (model 3). Fluid intelligence did not show associations with FC in males. Townsend deprivation indices, level of education and birth order did not show associations with FC.

In sensitivity analysis (exclusion of cases with high refractive errors and poor VA), the coefficients presented in Tables 3 and 4 remained remarkably stable. An additional model did not show clear associations with other early life factors (including self-recalled birth weight, maternal age at birth, maternal smoking around birth, and breastfeeding status as a baby), and significant coefficients shown in the results section remained remarkably stable (data not shown, available on request).

## Discussion

In addition to developing an automated method to quantify OCT-derived FC, this is the first study to assess FC associations with sociodemographic and ocular factors at scale. Ethnic differences in FC were marked, with black, Asian, Chinese, mixed, or other ethnic groups having flatter foveal curvatures when compared with whites. Ethnic differences were systematically larger in men than in women. In both sexes, SE, corneal astigmatism, and CPRT showed graded inverse associations with FC whereas VA showed graded positive associations; and regression coefficients were remarkably similar in men and women. Increase in income (in males), MC, and age (in females) showed an association with steeper FC. Different patterns of associations were observed between males and females and in different directions with increasing age. These associations, except for MC in males, held after adjustment and removal of participants with extreme refraction and poor visual acuity. Our findings suggest associations between sociodemographic and ocular factors with FC and provide evidence of novel associations with foveal morphology.

### Ethnicity

Reports on FC variations across ethnic groups have shown unclear associations.^18^^,^^19^^,^^29^^–^^31^ Wagner-Schuman et al.^19^ evaluated OCT-derived foveal morphology of 180 eyes of 90 patients, and despite finding significantly deeper and wider foveal diameters in black patients when compared to whites, the differences in foveal steepness were not significant. Similarly, an average of 1.05° smaller foveal slope was reported in Ghanaians versus whites by Zouache et al.,^18^ but the difference failed to reach statistical significance. Macular pigment has been suggested to play a determinant role in foveal morphology. Black and south Asian ethnic groups have shown higher macular pigment densities when compared to whites; however, no differences in foveal slope have been identified in a more recent study.^31^ Furthermore, hypomorphic *TYR* R402^32^ and S192Y^32^^,^^33^ alleles have recently been identified to be associated with foveal morphology (smaller fovea diameter, smaller foveal avascular zone area, and increased retinal thickness). We demonstrate substantial ethnic differences in FC, with flatter FC found in non-white individuals when compared with whites. Interestingly, the association with ethnicity was strengthened after adjustment and exclusion of extreme refraction and VA worse than 6/7.5. Macular thickness has been found to vary across ethnicities, with non-white individuals having a thinner central retina.^17^^,^^19^^,^^34^^–^^37^ Our findings of thinner foveas in non-whites versus whites add to the literature by quantifying the CPRT rather than an average measurement of the central subfield that can be affected due to pit curvature (Supplementary Fig. S4).^37^

### Sex

Evidence in the literature about sex differences in FC (slopes) has shown unclear results. Zouache et al.^18^ compared OCT-derived foveal morphology of 87 Ghanaian and 37 white patient eyes, finding flatter average foveal slope in females when compared with males (*P* < 0.001); nevertheless, the difference was only observed in the Ghanaian ethnic group.^18^ In contrast, data derived from other studies have reported foveal slopes to be independent of sex.^16^^,^^17^^,^^19^ In our study, males showed a steeper FC when compared to females, evidencing a clear association with this anatomical parameter.

A positive correlation of height with macular thickness has been reported.^38^ In our study, height differences between sex were statistically significant, and a significant positive association with FC was found in this study (Tables 3 and 4).

### Age

Few studies have investigated age-related changes in fovea morphology.^6^^,^^17^^,^^18^^,^^39^ An unexpected steeper FC association with increasing age that persisted after adjustment and exclusion of cases with extreme refraction and low VA was only observed in females in our study. In line with our findings, Nesmith et al.^17^ showed an increase in foveal asymmetry and a steeper slope with increasing age. In contrast, Zouache et al.^18^ and Tick et al.^39^ have found no associations between fovea morphology with age. Other analyses have not adjusted for age when evaluating FC.^19^ Vitreous degenerative changes,^40^ foveal cone density decline,^41^ rod structural changes with length increase in outer segments,^42^ and Müller cell^3^^,^^43^ decline with age (leading, in conjunction, to a reduction in CPRT with stable parafoveal retinal thickness) are possible underpinning factors for the increase in FC seen in females with age. This is further supported by a different pattern adjusted mean CPRT seen with age between males and females in our analysis (See Supplementary Fig. S4). Patel et al.^37^ found a biphasic association between central subfield macular thickness and age in UK Biobank participants with an increase in macular thickness from 40 to 59 years, but no further increase from 60 to 69 years. However, the authors report combined results for males and females. The reason for lack of association of age with FC in males is unclear.

### Ocular Factors

As shown in previous reports,^11^^,^^44^^,^^45^ the presence of a depression is not required for foveal cone anatomical or functional specialization. Inner and outer retinal specialization can proceed independently, to some extent, and evidence that foveal development continues after premature birth exists.^4^ Notwithstanding, it has been shown that foveal structural grading can predict future VA in children and infants with absent or poorly formed foveal depressions with presence of inner retinal layers.^15^^,^^46^^,^^47^ By showing flatter FC in eyes of healthy individuals with VA worse than 6/7.5, we provide further evidence of the visual significance of the FC in healthy eyes. Concepts of foveal development postulate an antiparallel sequential shifting of the inner and outer retinal layers.^48^^–^^51^ From the model proposed by Springer and Hendrickson,^49^ which relied on histologic data and computerized simulations, the participation of mechanical factors (anteroposterior compression by IOP and lateral stretching by ocular growth) was suggested. In our cohort, comprised of individuals with an age range of 39 to 70 years, we did not find an association of IOP with FC, a finding that could suggest that the IOP influence in the foveal neuronal layer organization is limited to early developmental stages. As a proxy for AL,^52^^–^^54^ refractive status expressed as SE was associated with FC, with myopic refractive status showing steeper foveas than hyperopic refractions. In the largest analysis of OCT-derived MC to date using multivariable multilevel regression (adjusting for age, sex, ethnicity, refractive error, IOP, VA, corneal astigmatism and fluid intelligence) Müller et al.^8^ (unpublished data, 2022) found that ethnicity and refractive error had the strongest association with MC (i.e., flatter MC with every D increase in refractive error, and steeper MC in black, Asian, and other ethnic groups when compared with whites). We have shown that steeper MC are associated with steeper FC. The effect size seen in females was almost double that observed in males after adjustment (models 2 to 4, Tables 3 and 4).

### Income

In the general population, socioeconomic disparities can be associated with health inequalities, morbidity, and mortality.^55^^,^^56^ This is the first study that reports socioeconomic associations with FC. Interestingly, every increase in the category of annual income was associated with steeper FC in males (model 3; *p* for trend 0.005), which persisted after exclusion of extreme refractions and VA worse than 6/7.5. Income associations with FC in females were not significant. Deprivation showed no significant associations (model 3).

Strengths of our study include its large sample size of 109,160 eyes with OCT-derived FC measurements from 63,939 patients. The breadth of sociodemographic, and ocular factors unprecedented on this scale, allow consistency of associations. Limitations of our study are as follows: first, AL was not available to perform OCT B-scan distortion correction. Therefore, as currently measured, FC could deviate in patients with different AL than the nominal AL given by the OCT platform. It is known that the parallel OCT B-scan assembly and image processing from imaging platforms can distort the spatial geometry of the true macular shape.^16^^,^^57^^,^^58^ Distortion-corrected morphological macular OCT studies to date have been undertaken using different analysis methods, mathematical models, and foveal landmarks definitions. In this context, it has been reported that foveal morphology remains largely independent of AL after distortion correction.^57^ Although others have found morphological differences (mainly foveal width) in corrected versus uncorrected OCT scans.^16^^,^^19^^,^^58^ It should be highlighted that our study encompasses data from single OCT imaging platform, and that horizontal raster OCT scans did not undergo any sort of spatial processing that would alter the retinal morphology (12 March 2019). Second, we did not undertake a retinal layer thickness analysis which could have provided further insight on the retinal layers responsible for these morphological, and functional changes. Third, it was cross-sectional, and firm conclusions on causal associations cannot be drawn. Fourth, we did not analyze the genetics of FC, and although genetic analyses associated with retinal thickness in healthy eyes have been reported,^33^^,^^59^ our study highlights the need to identify genetic variants associated with FC. With the current advances in machine learning, algorithms that assess OCT parameters will increasingly be deployed in clinical settings. In this context, ethnic differences in FC have important implications in the development of future algorithms and stresses the need for ethnically diverse datasets for training and testing, to avoid inequalities in outcomes.

## Conclusion

These findings highlight novel associations between OCT-derived FC and sociodemographic, VA, and ocular factors, with the greatest effect sizes in ethnicity. Our findings could represent the result of independent maturation or development of inner and outer retinal layers during development and suggest FC as a candidate marker to comprehensively assess the fovea in health and disease.

## Supplementary Material

Supplement 1
