# Supplementary material for: Foveal Curvature and Its Associations in UK Biobank Participants
Source: Invest Ophthalmol Vis Sci. 2022 Jul 28;63(8):26. doi: 10.1167/iovs.63.8.26 (PMC9344217; doi:10.1167/iovs.63.8.26)

Supplementary material

Machine learning model and model parameters.

Since the A* algorithm is prone to errors when handling images with poor contrast or abnormal brightness filled with random noise, we calculated the minimum retinal thickness and standard deviation (SD) for each B-scan to only include scans with thicknesses within 30 to 80 pixels (180 to 480 µm at an axial resolution of 6 µm/pixel), and SD ≤ 10 pixels (60 µm).
When we filtered out segmentation errors from A* algorithm, many hard examples were also excluded from the dataset. Therefore, the trained model was not robust enough at this point. In order to bolster this weakness, we retrained our model with additional hard examples. For this, we first processed 1,250 FDA data and excluded segmentation error. Next, we computed a sum of entropy for each obtained segmentation mask where each pixel has a probability value of being foreground. We then regarded examples with high entropy value as 'hard examples'. These hard examples were sorted in descending order, and the top 200 images were added to the original dataset for the training.
The Pyramid Parsing Network (PSPNet) with the ResNet18 backbone as our segmentation architecture model, was trained with a binary cross-entropy loss function using Adam optimizer. We chose a batch size of 8. The learning rate was initially set to 1×10^{-3}, and decay over each update was set to initial learning ratio divided by epochs. All inputs and outputs were cropped to 512 x 512 by setting y coordinates whose sum of pixel values has a maximum intensity to the center and then resized to 256 x 256. All images were normalized to a range between 0 and 1. During training, shadows and gaussian/speckle noises were randomly applied to the training data as well as basic data augmentations such as shift, flip, and rotation.
A total of 170,079 eye images were processed and 128x170,079 segmentation masks were generated by the segmentation network. Because the B-scans often include several vertically flipped images, we built a binary classification network with LeNet architecture that can identify flipped B-scans and unflip them automatically.

Fovea curvature extraction

We assumed that the center of the fovea had the smallest height between the upper and lower boundaries of the segmentation masks. We therefore used height maps generated from the two boundaries to detect the center fovea. First, the ILM and RPE boundaries were extracted by simply tracking the top and the bottom boundary for each generated segmentation mask. By mapping the distance between ILM and RPE boundaries for each location on the 128 B-scans, we obtained a 128x256 height map where each pixel represents the height of ILM-RPE. Next, we applied gaussian blur to the height map (Supplementary Figure 1A). We then binarized the filtered height map using Otsu's algorithm (Otsu, N., 1979. A threshold selection method from gray-level histograms. *IEEE transactions on systems, man, and cybernetics*, *9*(1), pp.62-66.). (Supplementary figure 1B). The resulting donut-shaped blob was then detected by a minimum circularity

threshold (Supplementary figure 1C). The center of the extracted blob [c_x, c_y] was then determined to be the center of the fovea.

Supplementary Figure 1.

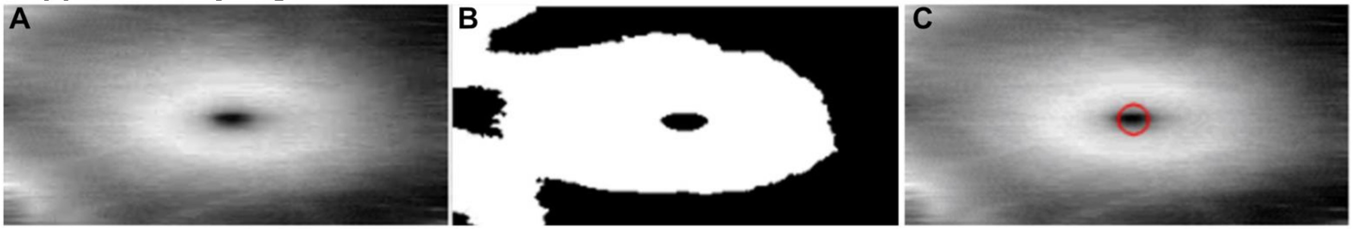

Foveal curvature validation

To validate the automated OCT-derived FC measurements, we divided eyes by FC tertiles. We then selected at random one eye per participant from each tertile, extracted the corresponding B-scan from the selected eye, and created a PSD file which consisted of one B-scan per tertile arranged at random (yielding a total of 10 independent sets with 3 B-scans, one from each curvature tertile). A file containing the B-scan ordered by fovea curvature, per set, was created as a CSV file and used for comparison. Two retina specialists with wide experience in OCT grading (AT, AO-B) were asked to classify each OCT B-scan, from flattest to steepest FC tertile, from each set. The human classification revealed perfect agreement when compared with tertiles derived from the automated quantification.

Supplementary Figure 2.

Representative example of polynomial fit to macular curvature. A convex macular curvature (left) and a concave macular curvature (right) are clearly evidenced on the image.

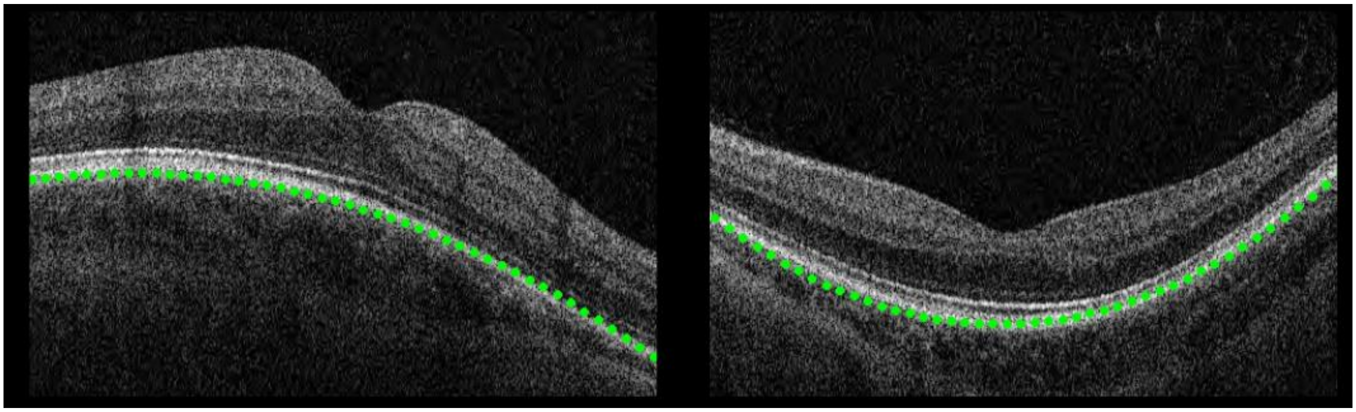

Supplementary Figure 3.
Top: foveal curvature quantification from central b-scans for each curvature tertile (from right to left, flattest to steepest quantified curvature measurements). Bottom: histogram of foveal curvature distribution by FC tertile.

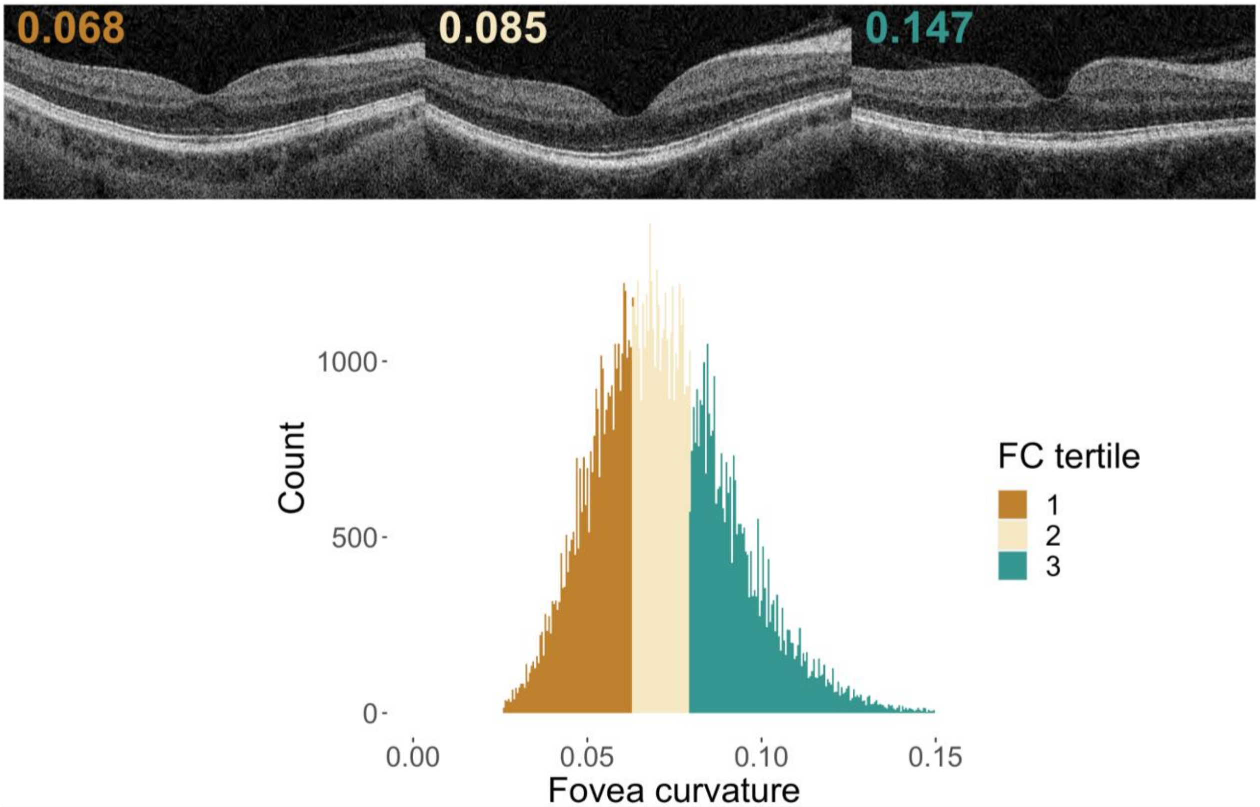

Supplementary table 1. Regression table for females with fovea curvature x 100 as dependent variable.

Characteristic	Females model 1*	Females model 2†	Females model 3‡
**Age (per decade)**	0.13 (0.11, 0.16); **6.8e-28**	0.16 (0.14, 0.19); **1.8e-34**	0.16 (0.14, 0.19); **9.4e-30**
**Ethnicity**			
White	1.00	1.00	1.00
Black	-1.1 (-1.2, -1.0); **5.1e-75**	-1.5 (-1.6, -1.3); **1.8e-120**	-1.4 (-1.5, -1.3); **2.3e-93**
Asian	-0.93 (-1.1, -0.80); **2.2e-44**	-1.2 (-1.3, -1.0); **1.4e-63**	-1.1 (-1.3, -1.0); **9.9e-48**
Other	-0.71 (-0.87, -0.55); **3.8e-18**	-0.90 (-1.1, -0.73); **4.4e-25**	-0.80 (-1.0, -0.62); **8.2e-18**
Mixed	-0.30 (-0.49, -0.11); **0.002**	-0.51 (-0.71, -0.31); **3.7e-07**	-0.47 (-0.68, -0.26); **7.5e-06**
Chinese	-0.31 (-0.59, -0.03); **0.032**	-0.63 (-0.92, -0.34); **2.5e-05**	-0.59 (-0.90, -0.28); **1.7e-04**
Prefer not to say	-0.84 (-1.2, -0.51); **6.9e-07**	-1.0 (-1.4, -0.67); **1.7e-08**	-1.0 (-1.4, -0.66); **9.5e-08**
Missing	-0.81 (-1.3, -0.37); **3.5e-04**	-0.88 (-1.4, -0.40); **3.1e-04**	§
**Height (per 5cm)**	0.08 (0.06, 0.09); **5.5e-24**	0.07 (0.06, 0.09); **2.4e-19**	0.07 (0.05, 0.08); **6.9e-16**
**Visual acuity (per 5 letters)**		0.03 (0.02, 0.03); **1.6e-16**	0.03 (0.02, 0.03); **1.4e-15**
**Spherical equivalent (per diopter)**		-0.08 (-0.09, -0.08); **6.4e-105**	-0.08 (-0.09, -0.07); **1.3e-96**
**Corneal astigmatism (per diopter)**		-0.14 (-0.16, -0.12); **3.2e-33**	-0.14 (-0.16, -0.12); **4.0e-32**
**Macula curvature (per 0.01)**		0.58 (0.46, 0.69); **1.4e-21**	0.55 (0.43, 0.67); **1.7e-19**
**Center point retinal thickness (per 10µm)**		-0.26 (-0.27, -0.25); **0.0e+00**	-0.26 (-0.27, -0.25); **0.0e+00**
**Fluid intelligence**			0.02 (0.01, 0.03); **9.8e-04**
**Annual income (Great British Pound)**			
Less than 18,000			1.00
18,000 to 30,999			0.01 (-0.05, 0.08); 0.701
31,000 to 51,999			0.02 (-0.05, 0.09); 0.589
52,000 to 100,000			0.02 (-0.05, 0.09); 0.605
Greater than 100,000			0.07 (-0.03, 0.17); 0.181
Prefer not to say			0.07 (0.00, 0.15); 0.055
Missing			-0.04 (-0.53, 0.45); 0.872
**Birth order**			
1			1.00
2			0.02 (-0.02, 0.07); 0.311
3			-0.05 (-0.13, 0.03); 0.259
4			-0.03 (-0.11, 0.06); 0.569
Missing			0.38 (-0.45, 1.2); 0.371

Bold p-values represent statistically significant results.
* Model 1: multilevel model adjusts for age, ethnicity, and height as fixed effects, and a random effect for person to allow for within person eye measurements (59,642 eyes of 35,097 patients).
† Model 2 adjusts as model 1 plus visual acuity, spherical equivalent, corneal astigmatism, macular curvature, and center point foveal thickness as fixed effects (54,489 eyes of 32,564 patients).
‡ Model 3 adjusts as model 2 plus deprivation, higher education, fluid intelligence score, annual income, and birth order as fixed effects (53,135 eyes of 31,727 patients).
§ No missing data on ethnicity on this model.

Supplementary table 2. Regression table for males with fovea curvature x 100 as dependent variable.

Characteristic	Males model 1*	Males model 2†	Males model 3‡
**Age (per decade)**	-0.06 (-0.08, -0.03); **8.4e-05**	-0.02 (-0.05, 0.01); 0.168	-0.01 (-0.04, 0.02); 0.466
**Ethnicity**			
White	1.00	1.00	1.00
Black	-1.2 (-1.3, -1.0); **6.0e-49**	-1.7 (-1.8, -1.5); **2.1e-88**	-1.6 (-1.8, -1.4); **5.2e-68**
Asian	-0.71 (-0.86, -0.56); **7.4e-21**	-1.1 (-1.2, -0.92); **1.7e-41**	-1.1 (-1.2, -0.90); **2.3e-34**
Other	-0.57 (-0.78, -0.36); **1.5e-07**	-1.0 (-1.2, -0.74); **1.5e-17**	-0.94 (-1.2, -0.71); **7.2e-15**
Mixed	-0.41 (-0.69, -0.12); **0.005**	-0.77 (-1.1, -0.47); **3.8e-07**	-0.73 (-1.0, -0.43); **3.0e-06**
Chinese	-0.68 (-1.1, -0.29); **6.5e-04**	-1.1 (-1.5, -0.65); **4.5e-07**	-1.1 (-1.5, -0.65); **8.9e-07**
Prefer not to say	-0.26 (-0.61, 0.09); 0.150	-0.25 (-0.62, 0.11); 0.168	-0.18 (-0.57, 0.21); 0.372
Missing	0.00 (-0.51, 0.52); 0.986	-0.09 (-0.61, 0.44); 0.749	-1.1 (-3.8, 1.6); 0.437
**Height (per 5cm)**	0.08 (0.06, 0.10); **4.3e-19**	0.07 (0.05, 0.09); **3.4e-15**	0.06 (0.05, 0.08); **4.2e-12**
**Visual acuity (per 5 letters)**		0.02 (0.01, 0.03); **3.5e-07**	0.02 (0.01, 0.03); **8.4e-07**
**Spherical equivalent (per diopter)**		-0.07 (-0.08, -0.07); **5.3e-53**	-0.07 (-0.08, -0.06); **5.6e-50**
**Corneal astigmatism (per diopter)**		-0.12 (-0.15, -0.10); **3.6e-20**	-0.13 (-0.15, -0.10); **5.3e-20**
**Macula curvature (per 0.01)**		0.27 (0.14, 0.41); **1.0e-04**	0.28 (0.14, 0.42); **1.2e-04**
**Center point retinal thickness (per 10µm)**		-0.29 (-0.30, -0.28); **0.0e+00**	-0.29 (-0.30, -0.28); **0.0e+00**
**Fluid intelligence**			0.00 (-0.02, 0.01); 0.559
**Annual income (Great British Pound)**			
Less than 18,000			1.00
18,000 to 30,999			0.11 (0.03, 0.19); **0.010**
31,000 to 51,999			0.10 (0.02, 0.18); **0.021**
52,000 to 100,000			0.13 (0.04, 0.22); **0.004**
Greater than 100,000			0.16 (0.05, 0.28); **0.005**
Prefer not to say			0.01 (-0.10, 0.12); 0.857
Missing			0.22 (-0.37, 0.81); 0.459
**Birth order**			
1			1.00
2			0.01 (-0.04, 0.06); 0.728
3			0.06 (-0.04, 0.16); 0.216
4			-0.05 (-0.15, 0.06); 0.375
Missing			-0.39 (-1.3, 0.50); 0.389

Bold p-values represent statistically significant results.
* Model 1: multilevel model adjusts for age, ethnicity, and height as fixed effects, and a random effect for person to allow for within person eye measurements (59,642 eyes of 35,097 patients).
† Model 2 adjusts as model 1 plus visual acuity, spherical equivalent, corneal astigmatism, macular curvature, and center point foveal thicknes as fixed effects (54,489 eyes of 32,564 patients).
‡ Model 3 adjusts as model 2 plus deprivation, higher education, fluid intelligence score, annual income, and birth order as fixed effects (53,13 eyes of 31,727 patients).

Supplementary table 3. Sensitivity analysis**.** Females.

	Model 1			Model 2			Model 3		
**Characteristic**	**Beta**	**95% CI**[1]	**p-value**	**Beta**	**95% CI**[1]	**p-value**	**Beta**	**95% CI**[1]	**p-value**
**Age (per decade)**	0.07	0.06, 0.09	**7.3e-28**	0.09	0.08, 0.11	**8.5e-37**	0.09	0.07, 0.11	**7.8e-30**
**Ethnicity**									
White	—	—		—	—		—	—	
Black	-0.54	-0.60, -0.47	**1.2e-59**	-0.72	-0.78, -0.65	**2.1e-92**	-0.70	-0.77, -0.62	**2.5e-75**
Asian	-0.48	-0.55, -0.40	**2.6e-36**	-0.59	-0.67, -0.51	**3.0e-49**	-0.55	-0.64, -0.47	**9.8e-37**
Other	-0.38	-0.47, -0.29	**1.0e-15**	-0.47	-0.57, -0.37	**1.4e-21**	-0.43	-0.53, -0.33	**4.6e-16**
Mixed	-0.14	-0.25, -0.04	**0.009**	-0.23	-0.35, -0.12	**4.0e-05**	-0.23	-0.34, -0.11	**1.4e-04**
Chinese	-0.11	-0.28, 0.06	0.212	-0.23	-0.41, -0.05	**0.014**	-0.21	-0.40, -0.03	**0.025**
Prefer not to say	-0.43	-0.63, -0.24	**1.1e-05**	-0.49	-0.69, -0.28	**2.7e-06**	-0.48	-0.71, -0.26	**1.9e-05**
Missing	-0.38	-0.65, -0.12	**0.004**	-0.43	-0.70, -0.15	**0.002**			
**Height (per 5cm)**	0.04	0.03, 0.04	**2.5e-16**	0.03	0.03, 0.04	**1.9e-14**	0.03	0.02, 0.04	**4.7e-12**
**Visual acuity (per 5 letters)**				0.01	0.00, 0.02	**0.002**	0.01	0.00, 0.02	**0.003**
**Spherical equivalent (per diopter)**				-0.05	-0.06, -0.05	**1.3e-85**	-0.05	-0.06, -0.05	**1.7e-79**
**Corneal astigmatism (per diopter)**				-0.07	-0.08, -0.05	**2.3e-19**	-0.07	-0.08, -0.05	**3.3e-18**
**Macula curvature (per 0.01)**				0.25	0.18, 0.32	**4.6e-11**	0.24	0.16, 0.31	**7.0e-10**
**Centre point foveal thickness (per 10µm)**				-0.11	-0.12, -0.11	**0.0e+00**	-0.11	-0.12, -0.11	**0.0e+00**
**Fluid intelligence**							0.01	0.00, 0.01	**0.004**
**Annual income (Great British Pound)**									
Less than 18,000							—	—	
18,000 to 30,999							0.00	-0.04, 0.03	0.804
31,000 to 51,999							-0.01	-0.04, 0.03	0.756
52,000 to 100,000							-0.01	-0.05, 0.03	0.621
Greater than 100,000							0.01	-0.04, 0.07	0.712
Prefer not to say							0.03	-0.01, 0.07	0.156
Missing							-0.08	-0.37, 0.20	0.567
**Birth order**									
1							—	—	
2							0.01	-0.01, 0.04	0.319
3							-0.02	-0.07, 0.02	0.291
4							-0.01	-0.06, 0.04	0.666
Missing							0.00	-0.50, 0.50	0.992

[1] CI = Confidence Interval

Multilevel models after exclusion of individuals with spherical equivalen refraction < - 6 D and > + 6 D, visual acuity < 80 ETDRS letters (worse than 6/7.5 Snellen, or worse than 0.1 logMAR equivalent). Model 1 adjusts for age, ethnicity, and height as fixed effects with a random effect per person to allow for right and left eye measurements. Model 2 extends model 1 by adjusting for visual acuity, spherical equivalent, corneal astigmatism, macula curvature, center point retinal thickness and fluid intelligence. Model 3 further adjusts for annual income, and birth order.

Supplementary Table 4. Sensitivity analysis. Males.

	Model 1			Model 2			Model 3		
**Characteristic**	**Beta**	**95% CI**[1]	**p-value**	**Beta**	**95% CI**[1]	**p-value**	**Beta**	**95% CI**[1]	**p-value**
**Age (per decade)**	-0.03	-0.04, -0.01	**6.1e-04**	0.00	-0.02, 0.01	0.649	0.00	-0.02, 0.02	0.995
**Ethnicity**									
White	—	—		—	—		—	—	
Black	-0.56	-0.65, -0.47	**1.6e-36**	-0.79	-0.89, -0.70	**3.7e-65**	-0.76	-0.86, -0.66	**2.7e-50**
Asian	-0.38	-0.46, -0.29	**1.9e-19**	-0.53	-0.62, -0.45	**1.0e-34**	-0.54	-0.63, -0.44	**9.5e-30**
Other	-0.25	-0.37, -0.14	**2.5e-05**	-0.44	-0.57, -0.32	**2.3e-12**	-0.42	-0.56, -0.29	**4.5e-10**
Mixed	-0.30	-0.45, -0.14	**1.9e-04**	-0.44	-0.60, -0.28	**7.4e-08**	-0.42	-0.59, -0.25	**8.3e-07**
Chinese	-0.42	-0.67, -0.16	**0.001**	-0.56	-0.82, -0.29	**3.2e-05**	-0.61	-0.88, -0.34	**9.0e-06**
Prefer not to say	-0.09	-0.29, 0.11	0.366	-0.10	-0.30, 0.10	0.339	-0.07	-0.29, 0.15	0.517
Missing	0.03	-0.26, 0.32	0.845	0.03	-0.27, 0.32	0.866	-0.53	-1.9, 0.85	0.452
**Height (per 5cm)**	0.04	0.03, 0.04	**3.4e-13**	0.03	0.02, 0.04	**1.5e-10**	0.03	0.02, 0.04	**3.2e-08**
**Visual acuity (per 5 letters)**				0.02	0.01, 0.03	**2.5e-05**	0.02	0.01, 0.03	**4.4e-05**
**Spherical equivalent (per diopter)**				-0.05	-0.06, -0.04	**5.3e-50**	-0.05	-0.06, -0.04	**3.9e-47**
**Corneal astigmatism (per diopter)**				-0.07	-0.09, -0.06	**2.4e-16**	-0.07	-0.09, -0.06	**4.1e-16**
**Macula curvature (per 0.01)**				0.03	-0.05, 0.11	0.487	0.03	-0.05, 0.12	0.431
**Centre point foveal thickness (per 10µm)**				-0.13	-0.14, -0.13	**0.0e+00**	-0.13	-0.14, -0.13	**0.0e+00**
**Fluid intelligence**							0.00	-0.01, 0.01	0.994
**Annual income (Great British Pound)**									
Less than 18,000							—	—	
18,000 to 30,999							0.04	0.00, 0.09	0.080
31,000 to 51,999							0.04	-0.01, 0.08	0.108
52,000 to 100,000							0.06	0.01, 0.11	**0.014**
Greater than 100,000							0.08	0.02, 0.15	**0.010**
Prefer not to say							0.00	-0.06, 0.06	0.927
Missing							0.02	-0.33, 0.36	0.917
**Birth order**									
1							—	—	
2							0.01	-0.02, 0.04	0.437
3							0.04	-0.02, 0.09	0.168
4							-0.03	-0.09, 0.03	0.283
Missing							-0.18	-0.66, 0.29	0.453

[1] CI = Confidence Interval

Multilevel models after exclusion of individuals with spherical equivalen refraction < - 6 D and > + 6 D, visual acuity < 80 ETDRS letters (worse than 6/7.5 Snellen, or worse than 0.1 logMAR equivalent). Model 1 adjusts for age, ethnicity, and height as fixed effects with a random effect per person to allow for right and left eye measurements. Model 2 extends model 1 by adjusting for visual acuity, spherical equivalent, corneal astigmatism, macula curvature, center point retinal thickness and fluid intelligence. Model 3 further adjusts for annual income, and birth order.

Supplementary Table 5. Descriptive table of eye level characteristics stratified by ethnicity and sex (Mean values defined by 95% confidence interval).

FEMALES

Characteristic	White, N = 54,820[1]	Black, N = 1,650[1]	Asian, N = 1,252[1]	Other, N = 801[1]	Mixed, N = 583[1]	Chinese, N = 253[1]	Prefer not to say, N = 182[1]	Missing, N = 101[1]
Fovea curvature x 100	6.90 (6.89, 6.92)	5.81 (5.72, 5.89)	5.84 (5.75, 5.92)	6.07 (5.95, 6.19)	6.53 (6.37, 6.68)	6.51 (6.30, 6.71)	6.09 (5.87, 6.32)	6.14 (5.80, 6.47)
Visual acuity (in ETDRS letters)	84.85 (84.77, 84.93)	84.21 (83.75, 84.67)	83.91 (83.37, 84.45)	83.31 (82.59, 84.04)	85.24 (84.49, 85.99)	83.10 (81.95, 84.25)	82.77 (81.42, 84.13)	82.32 (80.24, 84.40)
Spherical equivalent (Dioptre)	-0.68 (-0.71, -0.66)	-0.88 (-0.99, -0.76)	-0.96 (-1.11, -0.81)	-0.72 (-0.88, -0.56)	-1.16 (-1.38, -0.94)	-2.65 (-3.04, -2.26)	-0.70 (-1.10, -0.30)	-0.44 (-1.00, 0.13)
Corneal astigmatism (Dioptre)	0.89 (0.88, 0.89)	0.81 (0.78, 0.84)	0.80 (0.76, 0.84)	0.91 (0.85, 0.96)	0.88 (0.82, 0.93)	0.87 (0.79, 0.94)	0.86 (0.77, 0.95)	0.95 (0.82, 1.07)
Macula curvature x 100	0.21 (0.21, 0.21)	0.24 (0.23, 0.24)	0.22 (0.22, 0.23)	0.23 (0.22, 0.23)	0.22 (0.21, 0.23)	0.26 (0.24, 0.29)	0.21 (0.19, 0.23)	0.21 (0.18, 0.24)
Centre point foveal thickness (in µm)	224.01 (223.85, 224.17)	208.11 (207.35, 208.88)	213.57 (212.63, 214.51)	214.99 (213.82, 216.16)	217.71 (216.38, 219.04)	217.25 (215.22, 219.27)	215.96 (212.94, 218.98)	220.53 (217.01, 224.06)

[1] Mean (95% CI)

MALES

Characteristic	White, N = 45,636[1]	Black, N = 1,068[1]	Asian, N = 1,192[1]	Other, N = 572[1]	Mixed, N = 311[1]	Chinese, N = 160[1]	Prefer not to say, N = 198[1]	Missing, N = 92[1]
Fovea curvature x 100	7.82 (7.80, 7.84)	6.72 (6.61, 6.84)	7.00 (6.89, 7.11)	7.26 (7.08, 7.44)	7.40 (7.18, 7.62)	7.01 (6.77, 7.26)	7.53 (7.27, 7.79)	7.86 (7.41, 8.32)
Visual acuity (in ETDRS letters)	85.49 (85.39, 85.58)	84.91 (84.32, 85.49)	85.93 (85.44, 86.42)	84.79 (83.97, 85.61)	86.62 (85.60, 87.64)	82.41 (80.65, 84.16)	83.54 (82.19, 84.89)	83.74 (81.78, 85.70)
Spherical equivalent (Dioptre)	-0.64 (-0.66, -0.62)	-0.82 (-0.93, -0.71)	-0.97 (-1.10, -0.84)	-0.87 (-1.05, -0.68)	-1.13 (-1.39, -0.86)	-2.68 (-3.16, -2.19)	-0.65 (-0.96, -0.35)	0.16 (-0.22, 0.54)
Corneal astigmatism (Dioptre)	0.81 (0.80, 0.81)	0.80 (0.76, 0.84)	0.75 (0.72, 0.78)	0.83 (0.77, 0.89)	0.83 (0.74, 0.91)	0.76 (0.69, 0.84)	0.76 (0.68, 0.83)	0.92 (0.76, 1.08)
Macula curvature x 100	0.22 (0.21, 0.22)	0.24 (0.23, 0.24)	0.21 (0.20, 0.22)	0.22 (0.21, 0.23)	0.22 (0.20, 0.23)	0.31 (0.28, 0.34)	0.20 (0.18, 0.22)	0.18 (0.15, 0.20)
Centre point foveal thickness (in µm)	230.88 (230.69, 231.06)	211.04 (210.05, 212.04)	219.14 (218.12, 220.16)	218.51 (217.11, 219.90)	220.67 (218.39, 222.94)	220.89 (218.36, 223.43)	228.42 (225.77, 231.07)	224.84 (220.36, 229.32)

[1] Mean (95% CI)

Supplementary Figure 4. Adjusted mean centre point foveal thickness by deciles of age broken down by sex. Adjusted means (Solid black dots), 95% confidence intervals (Vertical solid lines) and regression line (Dotted line) are from a multilevel model allowing for age, height, ethnicity and UK Biobank centre as fixed effects, and repeated foveal curvature measurement for each person.

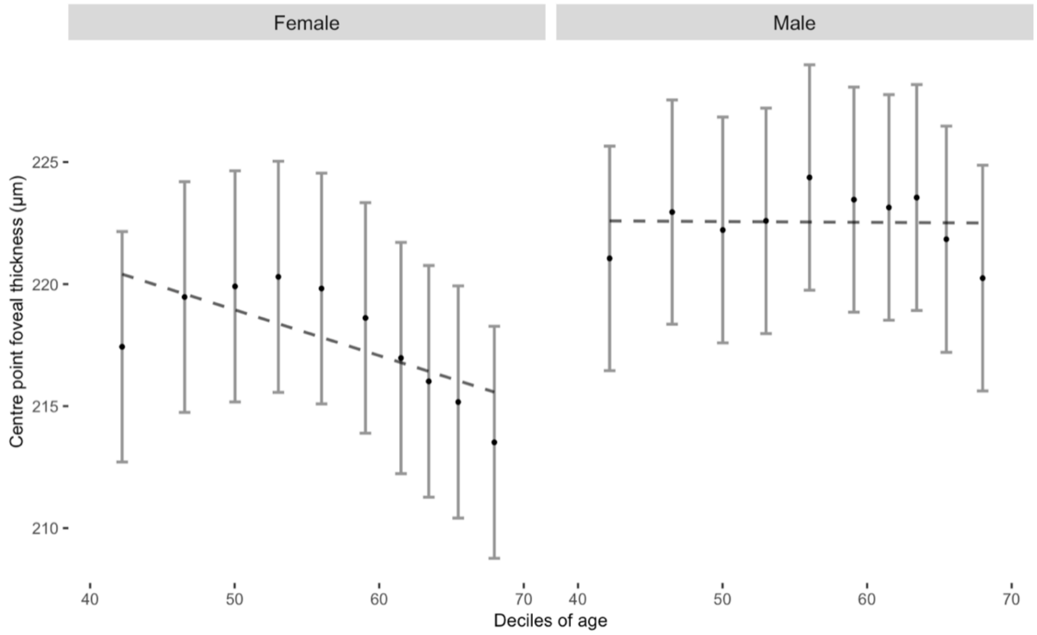

Supplement: Supplement 1 [file iovs-63-8-26_s001.pdf]
